# Regulation of Host Immune Responses against Influenza A Virus Infection by Mitogen-Activated Protein Kinases (MAPKs)

**DOI:** 10.3390/microorganisms8071067

**Published:** 2020-07-17

**Authors:** Jiabo Yu, Xiang Sun, Jian Yi Gerald Goie, Yongliang Zhang

**Affiliations:** 1 Integrative Biomedical Sciences Programme, University of Edinburgh Institute, Zhejiang University, International Campus Zhejiang University, Haining 314400, China; jiabo.17@intl.zju.edu.cn (J.Y.); 3170111563@zju.edu.cn (X.S.); 2 Department of Microbiology and Immunology, Yong Loo Lin School of Medicine, National University of Singapore, Singapore 117545, Singapore; e0004148@u.nus.edu; 3 The Life Sciences Institute, National University of Singapore, Singapore 117456, Singapore

**Keywords:** influenza, MAPK, respiratory viral diseases, pattern recognition receptors, inflammatory cytokines, innate immunity, adaptive immunity, cytokine storm

## Abstract

Influenza is a major respiratory viral disease caused by infections from the influenza A virus (IAV) that persists across various seasonal outbreaks globally each year. Host immune response is a key factor determining disease severity of influenza infection, presenting an attractive target for the development of novel therapies for treatments. Among the multiple signal transduction pathways regulating the host immune activation and function in response to IAV infections, the mitogen-activated protein kinase (MAPK) pathways are important signalling axes, downstream of various pattern recognition receptors (PRRs), activated by IAVs that regulate various cellular processes in immune cells of both innate and adaptive immunity. Moreover, aberrant MAPK activation underpins overexuberant production of inflammatory mediators, promoting the development of the “cytokine storm”, a characteristic of severe respiratory viral diseases. Therefore, elucidation of the regulatory roles of MAPK in immune responses against IAVs is not only essential for understanding the pathogenesis of severe influenza, but also critical for developing MAPK-dependent therapies for treatment of respiratory viral diseases. In this review, we will summarise the current understanding of MAPK functions in both innate and adaptive immune response against IAVs and discuss their contributions towards the cytokine storm caused by highly pathogenic influenza viruses.

## 1. Introduction

Viruses are major contributors of infectious diseases, causing significant morbidity and mortality worldwide [1]. Unlike bacteria, the life cycle of viruses involves host cell entries, viral genome replications, viral protein productions, assemblies of viral proteins and, lastly, the release from host cells [2,3]. As such, viruses are greatly dependent on host’s cellular machinery for virulence and survival. Fortunately, both innate and adaptive immunities have evolved and developed various ways to detect and eliminate virus and infected cells in response to viral infections. Surface viral proteins, such as the capsid and viral genomes, act as pathogen-associated molecular patterns (PAMPs), which are recognised by pathogen recognition receptors (PRRs) on immune cells [4]. They commonly include Toll-like receptors (TLRs), Retinoic Acid-Inducible Gene-I (RIG-I)-like receptors (RLRs) and Nod-like receptors (NLRs) that recognise both extracellular and intracellular viral-associated molecular patterns [5]. Upon recognition, adaptor proteins like MyD88 are recruited to activate downstream signalling, through a phosphorylation cascade, that leads to the activation of pro-inflammatory cytokines and Type I Interferons (IFNs) to induce an anti-viral state [5]. Likewise, in response to the selection pressure from the host immune response, the rapid yet error-prone replication of viruses allows them to constantly generate mutants that aid in the adaptation and evasion of host immunity [6]. This is clearly demonstrated in the never-ending persistence of global infections caused by the influenza A virus (IAV) every year due to its high rates of mutation [7].

### 1.1. Influenza A Virus

IAV belongs to the family of Orthomyxoviridae, and is the main etiological agent for influenza [8]. It is an enveloped virus containing eight segments of negative-sense, single-stranded RNA (ssRNA) with a total genome size of 13.6 kb that encodes for 10 different essential viral proteins [8]. There are four types of influenza viruses, namely influenza A, B, C and D, with influenza A and B having clinical relevance for humans [9]. However, unlike its counterparts, IAVs have an extensive range of animal reservoirs such as humans, pigs and birds, thus providing the possibility of cross-species transmissions [10]. IAVs express two major surface antigens, hemagglutinin (HA) and neuraminidase (NA), that play crucial antagonistic roles as virulence factors in mediating viral entry and release respectively [11]. Upon entering the host through the nasal cavities, the life cycle of IAVs begins after the attachment and invasion of respiratory epithelial cells via the recognition of terminal sialic acid (SA) residues on surface glycoconjugates, resulting in HA-mediated endocytosis [8,12]. The acidic environment within the endosome promotes conformational changes of the HA glycoprotein, exposing fusion peptides that facilitate pore formation on the endosomal membranes which allows for the viral genome to be released into the cytoplasm. Subsequently, viral RNAs translocate to the nucleus where they are transcribed and replicated for the synthesis of new viral genomes and proteins using the hosts’ cell machinery within the cytoplasm [3,8]. Lastly, viral genomic segments are packaged into virions before leaving the cell through the cleavage of SA using the surface antigen NA [3].

Both HA and NA aid in the classification of different influenza virus subtypes. In total, there are 16 HA (H1–H16) and 9 NA (N1–N9) subtypes, where each strain of IAV are named based on the various combinations and assortments of both glycoproteins within their host reservoirs [13]. For example, the 1918 H1N1, 1957 H2N2, 1968 H3N2 and the 1997 H5N1 pandemics had origins from non-human reservoirs of swine and birds [14,15,16]. The large repertoire and combination of surface glycoproteins ultimately contribute to the constant evolution of the virus through genetic mechanisms known as antigenic drift and shift [17]. Briefly, antigenic drift occurs as a result of random point mutations within the viral genome which are left unchecked due to the lack of a proof-reading mechanism in IAVs [17]. On the other hand, antigenic shift occurs when reassortment of viral RNA genomes between two or more virus strains generates a completely new viral strain, with pandemic potential due to novelty in its antigenic patterns [17]. Hence, these two processes contribute to variations within the HA and NA glycoproteins to differing extents, ultimately increasing the viral host range and evasiveness from detection by the host immune system. Consequently, antigenic drift is responsible for sporadic outbreaks of seasonal influenza epidemics annually, where constant surveillance is needed to keep influenza vaccines current [9]. Conversely, antigenic shift contributes to large scale influenza pandemics, although at a much less frequency. For instance, swines are susceptible to both avian, swine and human influenza viruses, and are prime vessels for genetic reassortment and recombination, which contributes to pandemics like the H5N1 avian influenza and the H1N1 swine influenza pandemics [18].

### 1.2. Host Immunity against IAVs

While IAVs commonly cause upper respiratory infections of moderate severity, infections of the lower respiratory tract, while rare, may lead to pneumonia with complications, like acute respiratory distress syndrome (ARDS), or even death from respiratory failure [19,20]. PAMPs of IAVs, such as ssRNA, are recognised by the PRRs: RLRs and TLRs, of epithelial and tissue-resident immune cells within the respiratory tract and lungs [12,21,22]. This activates a cascade of signal transduction pathways that induces the production of cytokines and chemokines, which promotes the recruitment and infiltration of innate immune cells like neutrophils and monocytes from the peripheral blood circulation to the site of infection within the pulmonary milieu to remove the virus and viral-infected cells [12,23,24]. One such example is the Type I Interferons (IFNs), which play crucial roles in the induction of the antiviral response [25]. IFNs can stimulate the expression of a large number of genes collectively known as the IFN-stimulated genes (ISGs) to induce an antiviral state in neighbouring uninfected cells [26]. Coupled with the recruitment of innate leukocytes to the site of infection, this causes both local and systemic inflammation that enhances the antiviral immune response in an effort to eliminate the IAVs.

When the innate immune defences fail to clear the infections established by IAVs, the adaptive immunity kicks in in a bid to clear the infection using antigen-specific cytotoxic T-cells and neutralizing antibodies from plasma cells. Naïve CD4^+^ and CD8^+^ T-cells are primed for differentiation into effector cells by professional antigen-presenting cells (APCs), like dendritic cells (DCs) and macrophages, through the major histocompatibility complexes (MHCs). For instance, naïve CD8^+^ T-cells differentiate into CTLs upon recognition of IAV-peptides presented on MHC I molecules [27,28,29]. They perform direct killing of IAV-infected cells through the production of cytotoxic granules containing perforins and granzymes, resulting in pore formation on the membrane and induction of apoptosis [30,31]. In contrast, CD4^+^ T-cells assist the immune response through the secretion of various soluble factors including IL-2 and IFNγ, as well as the expression of surface molecules including the CD40-CD40L axis for cell-cell interactions [32,33,34,35]. CD4^+^ T-cells are also pivotal in the activation of B-cells and high-affinity antibody production, the other arm of the adaptive immunity [36,37]. Accordingly, activated plasma cells produce IAV-specific antibodies for neutralisation, opsonisation or antibody-dependent cellular cytotoxicity (ADCC) against IAV-infected cells, promoting the degradation and clearance by phagocytes [38,39,40].

However, an unwarranted hyperactive immune response towards IAV infections may lead to immune-mediated respiratory damage on top of viral cytopathic effects, contributing to severe lung injuries which further complicate disease outcomes [41,42,43]. The activation of immune responses towards influenza infections, like most viral infections, are underpinned by the activation of several molecular pathways including the interferon regulatory factor 3 (IRF3), the nuclear factor kappa-light-chain-enhancer of activated B-cells (NFκB) and the mitogen-activated protein kinase (MAPK) pathways [44,45,46,47,48,49]. Dysregulation of these pathways may result in exaggerated immune responses such as immune cell overactivation and inflammatory cytokine overproduction known as the “cytokine storm”, which are commonly associated with worsened disease outcome and are attractive targets for immunomodulatory therapies [50,51,52]. In fact, severe lung injuries as a consequence of dysregulated immune action are not only a feature of severe IAV infections, but are also commonly presented in other severe respiratory viral diseases such as the Severe Acute Respiratory Syndrome (SARS), Middle East Respiratory Syndrome (MERS), and the current Coronavirus Disease 2019 (COVID-19) [53,54,55]. While they vary in severity, infections from these viruses gives rise to common clinical and pathological presentations including distinct pulmonary lesions, onset of dyspnoea and the progression into ARDS, with the need for invasive mechanical ventilation and life support [56]. Hence, understanding the molecular mechanisms driving the regulation of important cellular signal transduction pathways in response to respiratory viral pathogens, such as IAVs, is imperative for further understanding of the pathogenesis and development of potential novel immunotherapies for severe respiratory viral diseases.

### 1.3. Mitogen-Activated Protein Kinases (MAPKs)

The MAPKs, a family of proline-directed, protein-serine/threonine kinases, are evolutionary conserved cellular regulators that convey extracellular signals in the form of phosphorylation cascades to elicit targeted intracellular responses [57]. Mammalian MAPKs consist of extracellular-signal regulated kinase 1 and 2 (ERK1/2), c-Jun *N*-terminal kinases/stress-activated protein kinases (JNK/SAPK), p38 and ERK5. Broadly, growth factors and mitogens activate ERK1/2 and ERK5, while cellular stress signals and inflammatory cytokines activate both JNK and p38 [58,59]. Upon extracellular stimulation, MAPKs are activated downstream of sequentially activated protein kinases: MAPK kinase kinase (MKKK) and MAPK kinase (MKK) [58,59]. For instance, IAV infection has been shown to activate the Raf/MEK/ERK pathway which is essential for viral production and ribonucleoprotein (RNP) exports [60,61]. These kinases regulate the activities of a number of cytosolic and nuclear proteins, thereby controlling various cellular activities including activation, proliferation, differentiation, effector functions and apoptosis [57]. As such, it is of no surprise that dysregulation of MAPKs often results in the development of immune-mediated diseases [62].

Highly pathogenic IAVs (HPIAVs), such as H5N1, are capable of manipulating crucial host cell signalling, including MAPKs, to induce an overabundant expression of the inflammatory mediators. Such excessive and uncontrolled release of pro-inflammatory cytokines results in the generation of the cytokine storm that often leads to acute lung injuries and ARDS, which is a common characteristic of severe respiratory viral diseases caused by HPIAVs or other viruses including SARS-CoVs [63]. A growing body of evidence demonstrates that MAPKs play important roles in both innate and adaptive immunity against IAV infections, contributing to the development of immune-mediated pulmonary pathology [64,65]. The roles of MAPK pathways on IAV infections have been reviewed earlier [64,66], but how they are regulated in immune response to IAV infection and their contributions towards the cytokine storm due to dysregulated signalling are much less. Understanding the regulatory roles of MAPKs in IAVs will not only be important for the development of immunotherapy against IAV infections, but will also shed light on the pathogenesis of other severe respiratory viral diseases, such as COVID-19. As such, the aim of this review is not only to provide updates on the roles played by ERK, JNK and p38 in various immune cells in the pulmonary system upon IAV infections, but also highlight the detrimental effects of the cytokine storm on pulmonary pathologies caused by aberrant signalling of MAPKs from infections by HPIAVs.

## 2. MAPKs and Innate Immunity to IAVs

The innate immunity is important for viral containment, and if possible, elimination, upon infection and plays a pivotal role in the subsequent induction and regulation of the adaptive immunity. When left unchecked, an excessive innate immune response contributes greatly to the development of respiratory pathologies such as ARDS [19,20]. Over the past few decades, considerable amount of research efforts have been devoted to uncover the mechanisms underlying the recognition and containment of IAVs by the innate immunity [67,68]. Of which, a number of studies have investigated the roles of virus-sensing receptors, generation of antiviral effector cells and the underlying molecular mechanisms related to MAPKs, which demonstrated the importance of MAPKs in innate recognition, activation of the IFN system, and expression of cytokines and chemokines in response to IAV antigens.

### 2.1. Innate Recognition of IAV Infection

Innate immunity against IAV infections is induced upon recognition of viral ssRNA by various PRRs at various subcellular locations during its replication process. The replication cycle of IAVs begins from the entry of host cells through HA-mediated endocytosis [8]. The acidic environment within the endosome promotes conformational changes of the HA glycoprotein, exposing fusion peptides that facilitate pore formation on the endosomal membranes which allows for the viral genome to be released into the cytoplasm. Subsequently, nuclear translocation occurs where the viral RNAs are transcribed and replicated for the synthesis of new viral genomes before nuclear export for translation of proteins using the hosts’ cytoplasmic and endoplasmic reticulum (ER)-associated ribosomes [8]. During this process, the viral genome is recognized by the host’s PRRs: RLRs in the cytoplasm, TLR3 and TLR7 in the endosome [69,70]. Activation of the PRRs by IAVs in the epithelial and immune cells residing in the respiratory tract results in the activation of the IRF3, NFκB and MAPK signal transduction pathways. This in turn regulates the expression of the Type I IFNs (IFNα and IFNβ), pro-inflammatory cytokines such as interleukin 6 (IL-6) and tumour necrotic factor α (TNFα), as well as chemokines like monocyte chemoattractant protein-1 (MCP-1) [12]. For instance, within the cytosol of infected cells, the 5′-triphosphate ends of the viral ssRNA are recognized by RIG-I, which results in its activation and translocation to the mitochondria to activate the mitochondrial antiviral signalling protein (MAVS) for downstream signalling [71,72,73,74]. On the other hand, viral genome in endosomes are detected by TLRs including TLR7, which transduces signals through the MyD88 adaptor protein to activate IRF7, MAPKs and NFκB for the expression of Type I IFNs and other pro-inflammatory mediators [74,75]. Interestingly, some existing evidence suggests that RLR and TLRs may not necessarily be indispensable in the activation of immune response against IAV infections. A recent study by Wu et al. showed that deficiency in RLR did not affect the survivability of C57BL/6 mice after lethal influenza infections [76]. An earlier study by Jeisy-Scott et al. also showed that the loss of TLR7 did not impact memory CD8^+^ T-cell response, and only moderately affected the development of B-cell adaptive immune memory [77]. IAV infections may also activate the NLRP3 inflammasome pathway [78,79]. ssRNAs of IAVs were shown to activate the NLRP3 inflammasome, leading to a lysosomal maturation and reactive oxygen species (ROS)-dependent production of IL-1β and IL-18 [80]. NLRP3 inflammasome may also be activated through the proton-specific Matrix-2 (M2) ion channel encoded by IAVs, which plays a role in the acidification of the virus-containing endosomes leading to membrane fusion and release of virions into the cytosol [81]. M2 localises to the Golgi upon infection, leading to the acidification of the Golgi compartment, which activates NLRP3 inflammasome and IL-1β production. Lastly, the virulence factor PB1-F2 of IAVs is also able to induce the production of IL-1β through a NLRP3- and Caspase-1-dependent pathway [82].

### 2.2. MAPKs and Expression of Inflammatory Mediators in Response to IAVs

Once influenza viruses are recognized by PRRs, inflammatory mediators, including cytokines, chemokines and other antimicrobial factors, are secreted by various types of cells, including epithelial cells, endothelial cells and monocytes/macrophages. Among the cells that are capable of secreting cytokines/chemokines, airway epithelial cells and tissue-resident alveolar macrophages are the major and immediate sources of inflammatory mediators in response to respiratory tissue assaults [83,84,85,86]. Apart from the induction of Type I IFN response, airway epithelial cells also secrete a number of cytokines, including IL-6, TNFα, granulocyte colony stimulating factor (G-CSF) and granulocyte macrophage colony stimulating factor (GM-CSF), which are all essential for anti-influenza immunity [87,88]. For instance, IL-6 drives the transition from innate to adaptive immunity, whereas TNFα amplifies cytotoxic activity and impairs viral replication [89,90]. Likewise, G-CSF and GM-CSF are required for the differentiation of myeloid cells, such as alveolar monocytes/macrophages in particular, promoting their effector functions and reducing influenza-mediated morbidity and mortality [91,92,93].

Chemokines secreted by airway epithelial cells and pulmonary macrophages recruit both innate and adaptive immune cells to the lung to amplify the immune response and release of cytotoxic and inflammatory factors [87]. CXCL8 recruits neutrophils to the lungs, whereas IP-10/CXCL10 and RANTES/CCL5 promotes the infiltration of monocytes, NK cells and T-cells from the peripheral circulation into the lungs [94,95,96,97]. Once recruited, these cells work specifically and cooperatively to control and eradicate the viruses from the airways and lungs. Neutrophils are the first few immune cells recruited to the site of infection, playing a crucial role in clearing virions or dead viral-infected cellular bodies [98,99]. As their effector functions hinge on the use of antimicrobial peptides and proteolytic granules, influenza viral particles can be quickly degraded and cleared. Alveolar macrophages on the other hand, are the major source of Type I IFNs which are essential for recruiting inflammatory monocytes to the lungs and generating an antiviral state during early viral infections [100,101]. Upon secretion, Type I IFNs bind to their respective receptors on neighbouring uninfected cells which, stimulating the expression of ISGs which ultimately induce a cell-intrinsic antiviral state, responsible for effective inhibition of IAV spreading and infection through various mechanisms [26]. The expression of Type I IFNs may be regulated by MAPKs through the regulation of IRF3 or direct transcription of IFN genes. It is known that JNK regulates IFNβ expression through the activating protein-1 (AP-1) transcription factor in response to IAV infections [102]. ERK, JNK and p38 were also shown to increase the expression of heme oxygenase-1 (HO-1) through the induction of nuclear factor erythroid 2-related factor 2 (Nrf2), thereby promoting the expression of Type I IFNs to suppress IAV infections [103,104]. However, further studies are required to further establish such novel mechanisms of MAPKs in innate immunity against IAVs.

In addition to the expression and action of Type I IFNs, activation of the MAPKs in these cells during IAV infection also regulate the expression of inflammatory mediators. Indeed, in human bronchial epithelial cells, IAV infection resulted in the activation of JNK, p38 and ERK. Inhibition of JNK and/or p38, but not ERK, led to reduced expression of RANTES/CCL5, indicating that JNK and/or p38 activation is required for chemokine expression in response to IAV infection in airway epithelial cells [105]. This is further supported by a study analysing the transcriptomic profile of human bronchial epithelial cells in response to IAV infection. The study demonstrated that among 165 upregulated genes, 29 genes (about 17.5%) were regulated by JNK and/or p38 [106]. These genes are involved in various cellular activities including antiviral activity such as MX1, antigen presentation including HLA-A and HLA-C, cell adhesion such as ICAM-1, inflammation such as IL-6 and apoptosis such as CASP10 [106], demonstrating the essential and diverse functions of these kinases in airway epithelial cells in response to IAV infections. In monocytes/macrophages, MAPKs are activated temporally in response to IAV infections. In murine monocytic cells, the influenza virus X-31 induced the activation of JNK and ERK as early as 15 min post-infection, followed by p38 activation at 3 h post-infection [49]. The early activations of JNK and ERK were important for early cytokine/chemokine responses towards the infection, which was demonstrated by the wide spread inhibition of inflammatory mediators including TNFα, IL-6, MCP-1, MIP-1α/CCL3, RANTES/CCL5, KC/CXCL1, IP10/CXCL10, and G-CSF upon inhibition of JNK and/or ERK alone [49]. In human macrophages, inhibition of JNK and ERK by their specific inhibitors or by decoy receptor 3 (DcR3) inhibited the secretion of cytokines including TNFα, IL-6 and IFNα upon IAV H1N1 infection [107]. Furthermore, it was found that sesamin, a natural compound isolated from the Thai medicinal plant *Sesamum indicum,* inhibited both pro-inflammatory cytokines IL-1β and TNFα in human peripheral blood mononuclear cells, by inhibiting the activation of JNK, p38 and ERK in response to H1N1 influenza infections, in a study using a combinatorial screening and computational approach [108].

The importance of MAPKs in host innate inflammatory responses against IAV infections was confirmed by in vivo studies using animal models. It was found that DcR3-transgenic mice or mice administrated with DcR3 recombinant protein had reduced disease severity and lethality upon influenza H1N1 virus infection, with reduced expression of TNFα, IL-6 and IFNα in the lungs, which was associated with reduced activation of JNK and ERK [107]. In addition, it has been shown that infections by the H9N2 influenza virus strain, which originated from swine hosts, in BALB/c mice resulted in inflammation and injury in the lung with production of pro-inflammatory cytokines including TNFα, IL-1β and IL-6. Consequently, inhibition of p38 by its specific inhibitor SB203580 caused decreased levels of TNFα, IL-1β and IL-6 and alleviation of lung injury [109]. Together, these studies demonstrate the importance of MAPKs in regulation of inflammatory mediator expressions and pulmonary inflammation in response to IAVs.

### 2.3. MAPKs and Cytokine Storm Induced by Highly Pathogenic Influenza Infections

**Figure 1:**
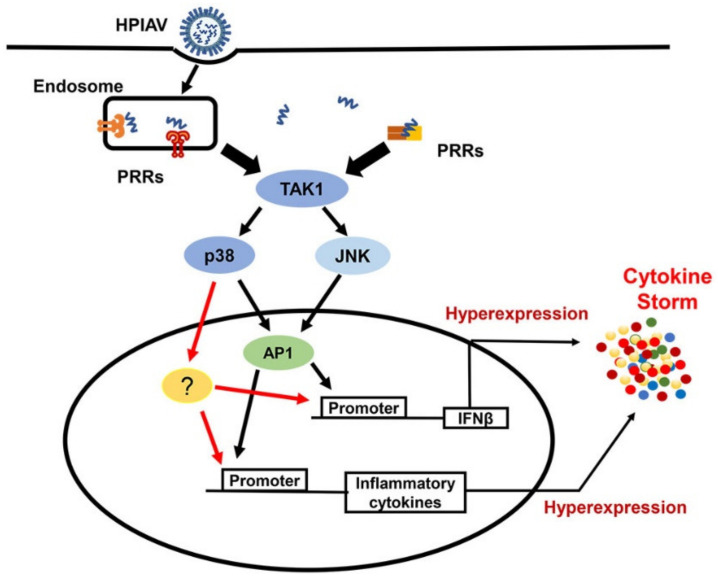
Pivotal Role of Mitogen-Activated Protein Kinases (MAPKs) in cytokine storm induced by highly pathogenic influenza virus infection. In response to infection by highly pathogenic influenza viruses (HPIAVs) such as H5N1 infection, PRRs, including RIG-I and TLR7 signalling, activates MAPKs, including JNK and p38, through TAK1. JNK activation contributes to hyper-expression of IFNβ and other inflammatory cytokines via transcription factor AP-1. p38, on the other hand, regulates more than 90% of inflammatory cytokine genes through AP-1 and other unknown factors. PRR—pattern recognition receptor; TAK1—TGF-beta activated kinase 1; TBK1—TANK-binding kinase 1; AP1—activator protein 1; ?—Other factors that have not been confirmed.

Highly pathogenic IAVs (HPIAVs), such as H5N1, are capable of manipulating crucial host cell signalling, including MAPKs, to induce an overabundant expression of the inflammatory mediators. Such excessive and uncontrolled release of pro-inflammatory cytokines, such as IL-1β, IL-6 and TNFα, results in the generation of the cytokine storm that often leads to acute lung injuries and ARDS, which is a common characteristic of severe respiratory viral diseases caused by HPIAVs or other viruses including SARS-CoVs [63]. The contribution of MAPKs to the development of the cytokine storm has been documented in numerous studies (Figure 1). In response to HPIAVs infection, such as infection by H5N1, which induces deregulated pro-inflammatory cytokine expression, inhibition of c-Jun, a target molecule of JNK and a component of AP-1, has been shown to suppress the expression of IFNβ and other cytokines, including IL-6 and TNFα. Thus, suggesting that uncontrolled activation of JNK induced by highly pathogenic IAV strains may contribute to the development of cytokine storm and severe lung pathologies [110]. Activation of p38 kinase has also been shown to play an important role in cytokine hyper-induction mediated by H5N1 virus in primary human macrophages [111]. p38 inhibition using the inhibitor SB203580 resulted in >80% reduction of IFNβ transcription and significant reduction of other cytokine expression, including IFN-λ1, TNFα, MCP-1 and IP-10/CXCL10, upon H5N1 infection without affecting IRF3 nuclear translocation, indicating that the regulation of these cytokine expressions by p38 is independent of IRF3 activation [111]. The reduction rate of IFNβ transcription by p38 inhibition was comparable to that caused by IRF3 knockdown, and the combination of both resulted in an additional 14% reduction of IFNβ transcription and further reduction of cytokine expressions [111]. Studies on cytokine expression in endothelial cells in response to highly pathogenic influenza virus infection further demonstrated the crucial regulating p38 in IAV-induced cytokine deregulation [112]. It was found that more than 90% of immune/inflammatory genes induced by HPIAVs, including H7N7 and H5N1, were dependent on p38. This kinase was not only able to directly regulate the transcription of IFNβ through its promoter but, was also able to control the expression of ISGs including MxA, OAS1 and IP10 through the phosphorylation of Tyr^701^ and Ser^727^ in STAT1 [112]. Furthermore, inhibition of p38 activation nearly abolished the hyper-induction of cytokines and protected mice from lethal infection of H5N1 [112]. Taken together, these studies demonstrated a central role of p38 kinase activation in the development of the cytokine storm after induction by HPIAVs, which may be targeted for the development of therapeutic interventions for severe influenza.

## 3. MAPKs and Adaptive Immunity to IAVs

When the innate immunity, as the first line of defence, fails to eliminate IAV infections during the first wave of response, the adaptive immunity takes over the role in clearing the virus in a second wave of response [12]. Cell-mediated and humoral immunity are two major components of adaptive immunity. Interaction with antigens, like surface glycoproteins HA and NA, by B-cell receptors (BCRs) leads to the activation and development of B-cells, which generates neutralising antibodies specific to infecting IAVs [113]. In addition, these anti-IAV antibodies can also indirectly eradicate the infected cells by triggering ADCC and the activation of the complement system [113]. However, antibodies are usually strain-specific, which provide limited and delayed protection in the outbreaks of new epidemic and pandemic strains due to antigenic drift or shift [114]. In contrast to antibody-mediated immunity, the larger repertoire of T-cell-mediated immunity may provide a wider breadth of defence against the highly evolving strains of IAVs, due to cross-reactivity across strains [115]. Nevertheless, it is imperative for both B-cells and T-cells to complement each other for an effective adaptive immune response. Accumulating evidences have also revealed that MAPK signalling is involved in T-cell immune responses to IAVs.

### 3.1. Dendritic Cells Bridge Innate and Adaptive Immunity

Dendritic cells, a group of specialised APCs, mediates the transition between the innate immunity to adaptive immunity during infection [12,29,104]. Immature DCs reside in the periphery of the respiratory tract and the lungs including airway epithelial tissues and alveolar spaces, where they detect inhaled pathogens via PRRs. Following antigen uptake, DCs migrate from the airway and the lungs to draining lymph nodes, where they present processed IAV antigens to T-lymphocytes to activate pathogen-specific CD4^+^ and CD8^+^ T-cells, respectively, to trigger adaptive immunity [29]. CD4^+^ T-cells are a distinct cell type involved in immune response towards IAVs. Depending on the stimulatory signals, they differentiate into different subtypes of T-helper cells and regulatory T-cells that are involved in cytokine production, cellular immune response regulation and activation of humoral immunity [37,116,117]. Activated by dendritic cells, CD8^+^ cells undergo proliferation and differentiation into cytotoxic T-cells (CTLs). CTLs reduce the expression of CCR7 and increase the expression of CXCR3 and CCR4 for migration towards the site of infection, where they recognise and kill infected cells through the release of cytotoxic granules containing perforins and granzyme B, and the induction of apoptosis by Fas/Fas ligand interactions [12,117,118]. They also produce pro-inflammatory cytokines which attenuate viral replication. Unlike humoral immunity mediated by B-cells, which are strain specific, CTLs target the highly conserved IAV peptides derived from influenza viral proteins, which allow them to display high cross-reactivity across different IAV strains [115,119].

### 3.2. The Significance of MAPK in Adaptive Immunity Against IAVs

**Figure 2:**
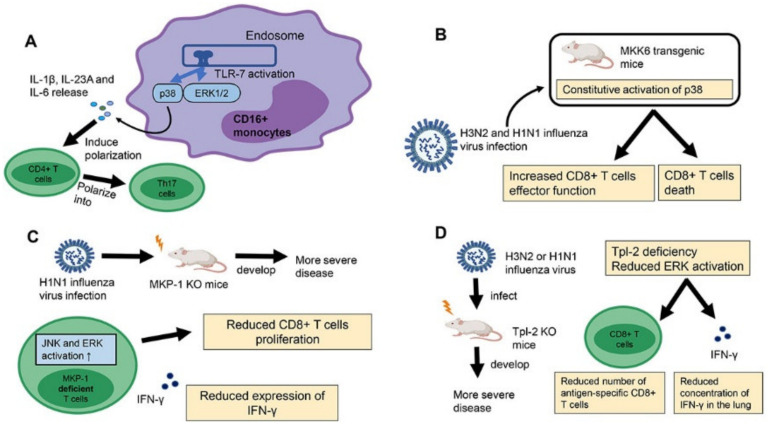
Regulatory function of MAPKs in influenza A virus (IAV)-specific T-cell responses. (**A**) Upon IAV infection, TLR7 signalling activates p38 and ERK1/2 in human CD16^+^ monocytes. Consequently, p38 and ERK1/2 activation induces expression of IL-1β, IL-23A and IL-6, which selectively polarize CD4+ T-cells into T_H_17 cells. (**B**) Constitutive p38 activation in MKK6 transgenic mice promotes CD8^+^ T apoptosis in response to H3N2 and H1N1 influenza virus infection, leading to reduced number of IAV-specific CD8+ T-cells. However, IAV-specific CD8^+^ T-cells enhanced effector function and the ability of virus clearance. (**C**) Activation of JNK and ERK is increased in MKP-1 deficient T-cells, associated with reduced antigen-specific CD8^+^ T-cells in the lungs and reduced IFN-γ expression by T-cells. Consequently, MKP-1 KO mice developed a higher severity of disease in response to H1N1 influenza virus infection. (**D**) Tpl-2 is a MAPK kinase kinase which activates ERK through MEK1/2. Compared to WT mice, the Tpl-2 KO mice developed a higher severity of the disease upon H3N2 or H1N1 influenza virus infection due to reduced number of influenza-specific CD8^+^ T-cells and reduced IFN-γ expression in the lung.

As innate immunity initiates and specifies adaptive immunity, it is not surprising that MAPKs regulate adaptive immunity against IAVs through the regulation of APCs. For example, it has been shown that IAV infection induces the expression of T_H_17-polarizing cytokines, including IL-1β, IL23A and IL-6 in human CD16^+^ monocytes, through TLR7-mediated p38 and ERK1/2 activation (Figure 2A) [120]. Consequently, TLR7 ligand-polarized human CD16^+^ monocytes preferentially drive naïve CD4^+^ T-cells to differentiate into T_H_17 cells characterized by high expression of IL-17A and RORC.

It has also long been known that MAPKs regulate T-cell activation, proliferation, differentiation, effector function and cell death [121]. However, there are limited evidence on the role of these kinases in regulation of IAV-specific T-cell responses, perhaps due to the fact that mice deficient in any of the three MAPK pathways are embryonic lethal [122,123,124]. However, studies using mice with genetically modified MAPK regulators provided important information on the regulatory function of MAPKs in T-cell responses to IAVs. A study using constitutively activated MKK6 transgenic mice, a p38-specific kinase, provided evidence of p38 in regulation of IAV-specific CD8^+^ T-cell response (Figure 2B) [121]. In response to H3N2 and H1N1 influenza virus infection, MKK6 transgenic mice had reduced accumulation of CD8^+^ T-cells in the lung compared to wildtype (WT) mice due to increased cell death. However, the transgenic mice were able to clear the virus more rapidly than WT mice, which was attributed to the increased CD8^+^ T-cell effector function for viral clearance. Therefore, while activation of p38 in IAV-specific CD8+ T-cells may improve antiviral immune responses through the production of IFNγ, the accompanying reduction in CD8^+^ T-cell numbers may reduce the overall effectiveness of the response.

MAPKs are negatively regulated by a family of protein phosphatases known as the MAPK phosphatases (MKPs), or also known as dual-specificity phosphatases (DUSPs). It has been shown that mice deficient in MKP-1, a member of the MKP family, had impaired viral clearance and poorer disease outcome compared to WT mice in response to IAV H1N1 PR8 virus infection (Figure 2C) [125]. The poorer disease outcome in MKP-1 KO mice was associated with reduced influenza-specific CD8^+^ T-cells in the lungs and reduced expression of IFNγ by both antigen-specific CD4^+^ and CD8^+^ T-cells compared to WT. The activation of JNK, and to a lesser extent, ERK, but not p38, was found to be increased in MKP-1 deficient T-cells compared to WT cells, which was associated with reduced proliferation and effector function in vitro and in vivo. This study indicated that activation of JNK and possibly ERK, but not p38, is required for the generation and effector function of both IAV-specific CD4^+^ and CD8^+^ T-cells for effective control of the infection.

A study on Tpl-2/MAP3K, a MAPK kinase kinase that activates ERK1/2 through MEK1/2, suggested a role of ERK1/2 in regulation of IAV-specific CD8^+^ response (Figure 2D) [126]. In response to H3N2 or H1N1 influenza infection, Tpl-2 KO mice developed an increase in severity of the disease compared to WT mice. Subsequent analysis showed that Tpl-2 was important for CD8^+^ T-cells response during infection and deficiency of Tpl2 resulted in reduced numbers of antigen-specific CD8+ T-cells and reduced concentration of IFNγ in the lungs, which resulted in impaired viral clearance and increased disease severity. Interestingly, ERK, JNK and p38 have also been shown to play a role in reduced T-cell response to influenza vaccine in aging. Sestrin-mediated simultaneous activation of ERK, JNK and p38, but not by their specific kinases, underpins T-cell senescence in aging. Inhibition of such alternative activation of ERK, JNK and p38 in aged mice increased both CD4^+^ T-cell and B-cell response to influenza vaccination, suggesting that MAPKs may be targeted to improve influenza vaccine efficacy in the elderly. Together, these studies demonstrate the importance of proper regulation of MAPK activation in T cells in response to IAV infection for prevention of adverse disease outcomes.

## 4. Targeting the MAPKs for Therapy

Studies in recent years have shown progress in targeting the MAPK pathways as a potential therapy against IAV infections (Table 1). As mentioned previously, the antiviral flavonoid 6-demethoxy-4′-*O*-methylcapillarisin (DMO-CAP) was able to induce the phosphorylation of p38, JNK and ERK, which promoted the expression of Type I IFN through the Nrf2/HO-1 pathway that inhibited the replication of IAVs [103]. The effects of virus replication inhibition against drug-resistant strains of H1N1 and H3N2 through the induction of HO-1 by DMO-CAP in vitro was comparable to that induced by a potent HO-1 inducer, cobaltic protoporphyrin IX chloride (CoPP). Oxymatrine, a traditional Chinese medicine obtained from the sophora root, was also shown to inhibit the phosphorylation of both ERK and p38, but not JNK, and the subsequent reduction of IL-1β, IL-6, IL-8 and TNFα, suggesting its potential in alleviating the induction of the cytokine storm [127]. In addition, Oxymatrine was also able to reduce the induction of TLR/MyD88/TRAF6, PI3K/Akt and NFκB pathways. In their in vivo model, Oxymatrine reduced lung inflammation and viral titres which was comparable to the effects of Oseltamivir, leading to improvements in alveolar exudation, integrity of alveolar walls and alveolar haemorrhage. Another study also showed that Vemurafenib, a B-Raf^V600E^ inhibitor, hyperactivated the Raf/MEK/ERK pathway, while inhibiting both p38 and JNK [60]. In addition, Vemurafenib also inhibited PI3K signalling induced by EGF stimulation, and was effective at low concentrations against both H7N7 and H1N1 strains. The authors also observed that Vemurafenib treatment suppressed apoptosis induced by IAV infection which was independent of the hyperactivation of Raf/MEK/ERK. They attributed this observation to the reduced SAPKs inhibited by Vemurafenib, which led to reduced TNF-related apoptosis-inducing ligand (TRAIL) expression. Lastly, impaired MAPK function due to Vemurafenib was led to interference with viral protein synthesis, ultimately disrupting the replication cycle of IAVs. Taken together, the current literature provides evidence that MAPK pathways harbour adequate potential as therapeutic targets against IAVs.

## 5. Discussion and Future Perspectives

IAVs are a continuous threat to global public health due to the rapid emergence of new strains from antigenic shift and drift, limiting the efficacies of vaccines. During infection, various factors contribute to the complicated interactions between the IAVs and host. Host immune responses toward IAV infections are critical for disease outcome, and understanding the interaction between IAVs and host cellular signalling pathways, as well as the regulation of host immune responses towards IAV infections, are important for the development of effective vaccines and novel therapies against influenza infections. MAPKs are important signalling molecules, which are activated by host cell receptor signalling in response to IAVs and underpin various immune cell activities against the infection. Dysregulation of MAPK activation leads to aberrant immune response and immune-mediated respiratory pathology, contributing to the severity and unfavourable outcomes of the disease. While a large amount of knowledge on the regulatory roles of MAPKs in immune response to IAVs has been achieved, detailed mechanisms on the roles of each MAPK in innate and adaptive immunity against IAVs, leading to effective immune control of the infection or exaggerated immune responses causing respiratory damage, remain unclear. One possible reason for these knowledge gaps is the lack of suitable model systems to study specific function of MAPKs in regulation of function of various types of immune cells during IAV infection. Hence, future studies may benefit from the development of animal and cellular models of IAV infection to elucidate the specific function of each MAPK, in both innate and adaptive immunity, against IAV infection, and how cross-talks among different MAPKs and with other important signalling molecules, including NFκB and IRFs, are regulated. This newfound knowledge will be essential for targeting the MAPK pathways to develop effective and further improve therapies for influenza infections and other respiratory viral diseases, such as the current COVID-19 pandemic.

## Figures and Tables

**Table 1:** Summary of potential drugs that act on the MAPK pathway against IAV infections.

Drug	Mechanism of Action	Effects on IAV Infections	Reference
DMO-CAP	• Activates ERK1/2, p38 and JNK which promotes expression of Type I IFN through the Nrf2/HO-1 pathway	• Inhibits IAV replication	Zhong et al., 2019 [103]
Oxymatrine	• Inhibits phosphorylation of ERK and p38, but not JNK • Reduce induction of TLR/MyD88/TRAF6, PI3K/Akt and NFκB pathways	• Reduced production of pro-inflammatory cytokines IL-1β, IL-6, IL-8 and TNFα which alleviates lung inflammation and viral titres	Dai et al., 2018 [127]
Vemurafenib	• Hyperactivates Raf/MEK/ERK pathway while inhibiting p38 and JNK • Inhibits PI3K signalling	• Suppressed apoptosis due to reduced TNF-related apoptosis-inducing ligand (TRAIL) expression • Interference in viral protein synthesis	Holzberg et al., 2017 [60]
Berberine	• Downregulates ERK pathway	• Inhibits virus replication	Botwina et al., 2020 [128]
